# Addressing the pharmacy leadership crisis: Pharmacy student placements in Alberta

**DOI:** 10.1177/17151635231188339

**Published:** 2023-07-26

**Authors:** Annissa Fung, Anne Truong, Maria Anwar

**Affiliations:** A. Fung and A. Truong were doctor of pharmacy students at the time of writing in the Faculty of Pharmacy and Pharmaceutical Sciences, University of Alberta, Edmonton; M. Anwar is with Pharmacy Services, Alberta Health Services, Calgary, Alberta

## Introduction

A growing body of literature on the pharmacy leadership crisis highlights how pharmacists do not feel prepared to pursue leadership roles.^1^ Pharmacists feel little incentive to aim toward taking on leadership roles and do not feel confident in their leadership abilities.^2^ To address this crisis, leadership placements are becoming more accessible to students as opportunities to push their boundaries and inspire the spark to lead themselves and others in the profession. We touch upon the work done in Alberta to encourage students and preceptors to take part in a leadership placement, highlight leadership development activities and opportunities and discuss the perceived value to students and preceptors.

Leadership-focused placements are a relatively new option available for University of Alberta pharmacy students. From 2020 to 2022, 73 leadership placements were offered by Alberta Health Services and Covenant Health, and 54 students were matched to these placements. Leadership placements were first offered to allow interested students to practice the leadership principles that are taught in pharmacy school. There is a call to action to develop more leaders,^2,3^ but this cannot just be taught in a classroom. Interviewed leadership placement preceptors believe pharmacy students are the next generation of pharmacy leaders and that leadership skills are essential to succeed as health care professionals.

## Addressing the leadership crisis through leadership placements

In Alberta, students are matched in a lottery system based on how they rank their preferred placements. Before placements are chosen, preceptors collaborate with the University of Alberta Faculty of Pharmacy and Pharmaceutical Sciences Experiential Education team to share their biographies, background information, expectations and the potential benefits of ranking a leadership placement.

We interviewed leadership placement preceptors, student alumni and current students to ask why they chose to undergo a leadership placement and how it affected them. Reasons for choosing a leadership placement included building upon knowledge gained from past leadership experiences, understanding one’s leadership style, building relationships with established pharmacy leaders, applying leadership lessons presented didactically in school and an opportunity for personal development and growth. A study on the perceived value of leadership experiences for American year 2 ambulatory pharmacy residents conducted by Smith et al.^4^ echoed how exposure to leadership, relationship building and learning to manage and lead self and others increased residents’ interest in pursuing formal or informal leadership roles and added significant value to their professional career.

Throughout the 6- or 8-week experience, students are exposed to a wealth of environments as various preceptors offer leadership placements. Students engage in activities to develop their leadership skills, such as observing and facilitating meetings, executing projects, applying project and change management methodologies and other activities that align with the student’s learning goals and the University of Alberta Faculty of Pharmacy and Pharmaceutical Sciences’ Elective Placement evaluation criteria.

When asked about the perceived value of leadership placements, a recurring theme in the responses of leadership placement alumni was that during pharmacy school, there is a lack of exposure to the pharmacist’s role in the health care system beyond clinical work in the community and hospital. By participating in a leadership placement, students learned about nontraditional career paths available for pharmacists, such as those of directors, managers and clinical practice leaders. They also built relationships with pharmacy and health care leaders. Students described the experience as a deep dive into applying different professional development skills such as communication and time management skills outside a clinical setting.

Relationships formed with preceptors and other current pharmacy leaders also proved valuable to students beyond the placement. Students expressed how building relationship equity with peers and mentors inspired them to seek out higher-level leadership roles and helped them overcome challenges faced later on in their careers. Students learned from the mentorship and leadership styles of their preceptors, which helped influence how they lead their teams.

## Design of the leadership placements

In-person, remote or hybrid format leadership placements were offered and completed over a duration of 6 or 8 weeks. Some placements apply a peer-assisted learning (PAL) model or are co-precepted by multiple preceptors. PAL model placements are 2 or more students assigned to 1 preceptor or a co-precepting team (e.g., 2:1 or 3:1). Benefits of a PAL model are supporting student collaboration and knowledge sharing. Students were given an outline of the leadership placement, which is inspired by the LEADS (Lead self, Engage others, Achieve results, Develop coalitions, Systems transformation) framework.^5^ Content included readings and multimedia outlining the focus of each week that students are invited to reflect, discuss and integrate into practice in alignment with their personal learning goals. Students became more independent as the weeks progressed and demonstrated growth in various leadership skills, behaviours and capabilities. Preceptors supported students by connecting with them during daily activity prebriefings, reflective discussions and project meetings.

Students are required to create a learning plan that highlights their SMART (Specific, Measurable, Achievable, Realistic and Timely) goals, strategies to attain those goals, progress indicators and documented progress. Informal feedback is offered daily, while formal feedback is conducted at the midpoint and final. The learning plans shaped the placement by identifying students’ needs, measuring progress and addressing how to achieve their leadership goals.

**Figure 1:**
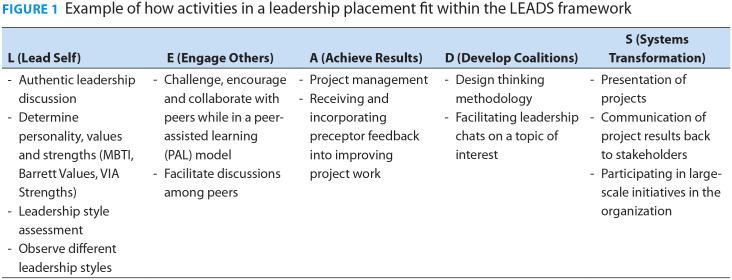
Example of how activities in a leadership placement fit within the LEADS framework

The placement began with students embarking on a journey of leadership self-discovery by completing personality assessments (VIA Strengths, Barrett Values Assessment, Myers-Briggs Type Indicator) to understand their strengths, values and leadership style. Following this, they began to work on projects while also attending meetings, networking, participating in facilitated discussions and other activities aligned with their learning goals (Figure 1). Throughout the placement, students learned and applied leadership concepts such as mitigating conflict, giving and receiving feedback, managing impostorism and learning about the importance of vulnerability, persuasion and influence. Students direct their development and lead their project work while immersed in a collaborative, safe and supportive learning environment with current pharmacy leaders and peers, which allows them to discover who they are as leaders and put their skills into practice.

A tailored approach to leadership placements helps create a psychologically safe space for students to be vulnerable, self-reflect, debrief and grow alongside their preceptors. Aspects of psychological safety such as trust, relationship building and educational alliance are essential for effective feedback. The principles and presence of psychological safety allow learners and preceptors to continue to challenge and grow together.^6^

Finally, students are evaluated using the University of Alberta Faculty of Pharmacy faculty rubric that is based on the Educational Outcomes of the Association of Faculties of Pharmacy of Canada. The rubric is general and organized into overarching domains of Care provider, Communicator, Collaborator, Manager, Advocate, Scholar and Professional.^7^ The generalizability of the rubric allows preceptors to incorporate students’ individual learning goals into the evaluation process.

## Discussion

How leadership is defined and measured may differ. The work by Schmeltzer et al.^2^ suggested that creating a dedicated set of pharmacy leadership competencies may encourage pharmacists to step up and lead. We argue that there are risks of measuring leaders to standardized competencies instead of encouraging them to lead authentically by living their values and harnessing their strengths. Our view aligns more with Blanchard’s theory of situational leadership and the belief that leaders are bred, not born.^8,9^ Individuals can learn how to become strong leaders by developing skills such as time management, self-awareness, emotional intelligence and the ability to communicate a vision and engage others to attain a common purpose.^2,10,11^

Each person can learn how to lead in a way that feels authentic to them. The concept of situational leadership states that leaders apply different skill sets and leadership styles depending on the circumstances. The same individual may tap into a directive leadership approach when aligning their team to a new organizational strategy or a supportive leadership approach when coaching a team member.^9^ As an individual’s leadership repertoire is not fixed, it would be unfair to assess them based on a rigid competency scale. Therefore, we favour the use of a framework to guide students and preceptors to discover how to lead in a way that feels authentic to them.

As the placement is self-directed, students experiment with what feels authentic to them throughout the placement. The onus is on the students to accept and seek out opportunities to grow. Students may be encouraged to learn outside their comfort zone; however, it is ultimately the student’s decision to determine what they want out of the placement. For example, the preceptor and student may discuss stretch goals at the beginning and midpoint to help with the growth of the student throughout the placement, but the student decides whether or not to challenge themselves to attain those goals.

Leadership concepts are broken down into learnable chunks over the placement. Through this, students’ leadership development is accelerated within the 6- or 8-week period as they are exposed to different types of leadership, pushed to apply and develop key leadership skills and prompted to reflect on who they are and what they stand for as leaders. The structure and evaluation process of leadership placements offered in Alberta moves away from “cookie-cutter” leadership, in alignment with the belief that leadership is bred, not born.^8,10,11^

## Conclusion

By offering leadership placements, we hope more students are interested in pursuing leadership development opportunities in the future. Placements aim to help students identify their own leadership identity in a safe, kind and supportive environment. With a unique placement structure, the creative environment allows for freedom within the framework and opportunities for students to build their own connections and community for their future practice. By emphasizing and encouraging students to embrace their authentic leadership style, we dismantle the idea of a “one-size-fits-all” approach to leadership that may be a barrier to many students.

Leadership placements serve as a space for students to develop the tools and skills to continue their ongoing leadership journey for years afterward. Following their placement, leadership placement alumni expressed the understanding that leadership development is an ongoing and iterative process. They aim to continue honing their leadership skills and incorporate what they have learned through the placement into future placements or pharmacy opportunities. Moreover, with increased confidence and ability to navigate challenging situations, students stated that they felt more prepared to move forward in their careers, pursue higher leadership positions and practice to their full scope. By building up a generation of strong and engaged leaders, leadership placements help to address the pharmacy leadership crisis one experience at a time.
